# Case Report of Low Virulence *Francisella tularensis* Presented as Severe Bacteremic Pneumonia

**DOI:** 10.1097/MD.0000000000003390

**Published:** 2016-05-13

**Authors:** Ting-Yi Su, Shian-Sen Shie, Ju-Hsin Chia, Ching-Tai Huang

**Affiliations:** From the Division of Infectious Diseases, Department of Internal Medicine, Chang Gung Memorial Hospital at Linkou, Chang Gung University College of Medicine (T-YS, S-SS, C-TH); Department of Laboratory Medicine, Chang Gung Memorial Hospital at Linkou (J-HC); and Department of Medical Biotechnology and Laboratory Science, Chang Gung University (J-HC), Taoyuan, Taiwan.

## Abstract

Tularemia is a zoonotic infection seen primarily in the Northern Hemisphere. It is caused by the bacteria *Francisella tularensis*. Although the ulceroglandular form of the disease is the more common manifestation of infection, *F tularensis* is known to cause pneumonia. *F tularensis* has two predominant subspecies, namely subsp. *tularensis* (type A) and subsp. *holarctica* (type B). Type B tularemia is considered to be much less virulent than type A and barely caused lethal disease and pneumonia.

We reported a case with a 68-year-old man immune-compromised patient diagnosed with bacteremic pneumonia engendered by type B tularemia with initial presentation of high fever, pneumonia with pleural effusion; the diagnosis was performed using 16S rRNA gene sequence analysis. The patient's fever, pneumonia, and pleural effusion were resolved with appropriate antibiotics for tularemia. This case involving severe bacteremic pneumonia in an immune-compromised patient is rare.

This case suggests that low virulence *F tularensis* should be included in the differential diagnoses of bacteremic pneumonia for endemic tularemia.

## INTRODUCTION

Tularemia is a potentially fatal, multisystemic disease in humans, and it is caused by *Francisella tularensis* and is primarily observed in the Northern Hemisphere including Europe, North America, and Asia. Ulceroglandular tularemia is the most common type of tularemia. Pneumonic tularemia occurs after exposure to aerosolized *F tularensis* particles or from hematogenous spread from a distal site; it has remained the most lethal form of tularemia.^1^*F tularensis* has two predominant subspecies, namely subsp *tularensis* (type A) and subsp *holarctica* (type B). Type B is much less virulent compared with type A and almost never causes lethal infection in humans.^2^ Tularemia pneumonia was a more invasive form of infection and more likely to be caused by *F tularensis* type A previously.^3^ In this report, we present a 68-year-old man Californian resident with tularemic bacteremic pneumonia caused by type B tularemia following treatment for 5 months with steroids for hypersensitivity pneumonitis. This case suggests that low virulence *F tularensis* should be included in the differential diagnoses of bacteremic pneumonia in immune-compromised hosts, particularly for those with endemic tularemia.

## CASE REPORT

In June, a 68-year-old man with a 3-day history of high fever was admitted to our hospital. The patient lived in a Californian metropolitan area for the past few decades and arrived in Taiwan 1 day before admission. He had been diagnosed with diabetes; dry cough, and progressive shortness of breath were observed for 6 months. Hypersensitivity pneumonitis was diagnosed after extensive studies involving high-resolution chest computed tomography (CT) and lung biopsy in addition to the exclusion of toxoplasma, rubella, cytomegalovirus, and herpes simplex virus type 1 and 2. Thereafter, a high dose of steroids with Prednisolone 60 mg per day was prescribed for 5 months.

**FIGURE 1:**
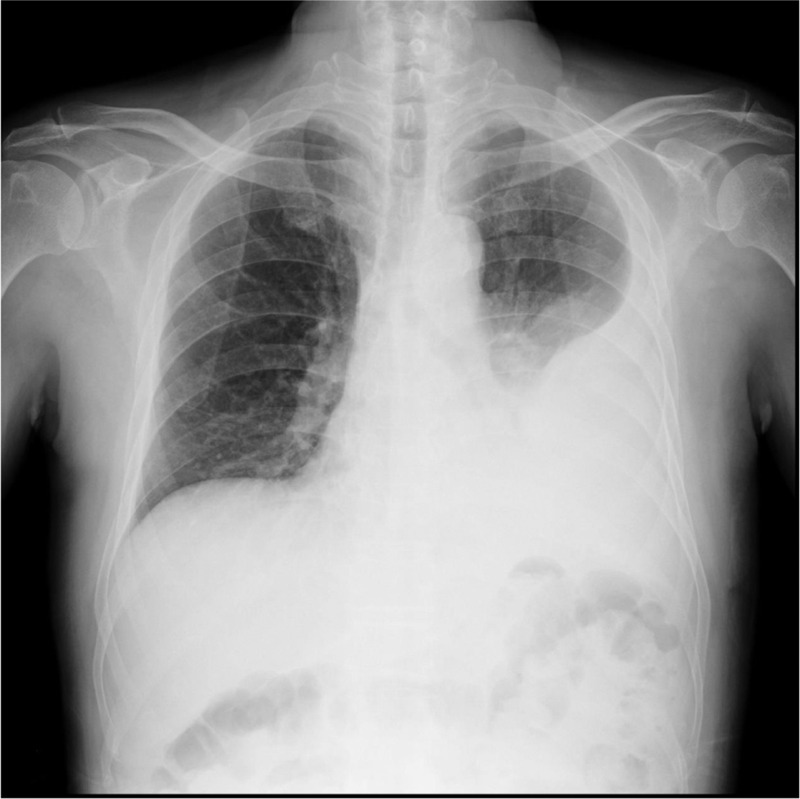
Admission chest radiograph: left lower lung consolidation and pleural effusion.

**FIGURE 2:**
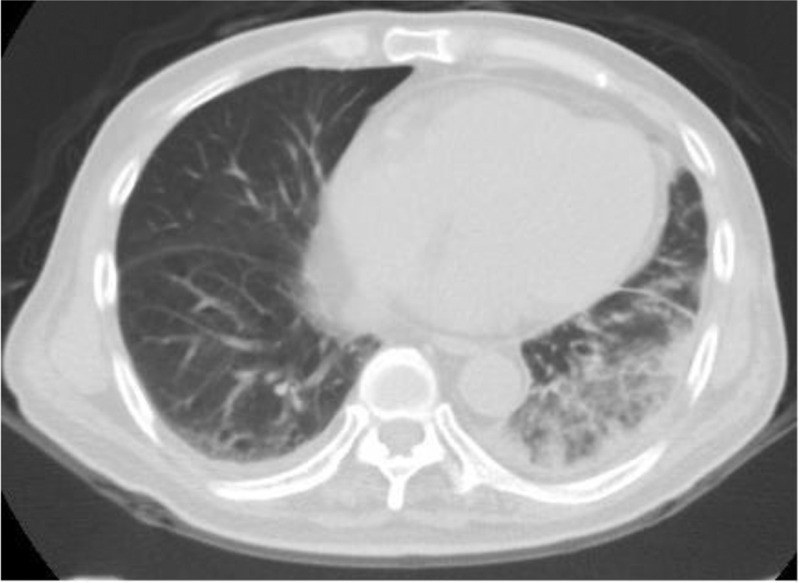
Initial chest computed tomography (CT): left lower lung air-bronchial ground with pleural effusion.

High fever developed 1 day before he arrived in Taiwan. During admission, laboratory examinations revealed leukocytosis and considerably elevated C-reactive protein levels (260.1 mg/L). A chest radiograph revealed left lower lung opacities (Figure 1). Despite the administration of empiric therapy with ceftriaxone plus clindamycin for community-acquired pneumonia, the fever persisted, and a left pleural effusion progressed rapidly. The pleural effusion was exudative, with a lymphocytic predominance. A drainage tube was introduced to alleviate severe dyspnea. Chest CT findings indicated pneumonia with a lung abscess (Figure 2).

**FIGURE 3:**
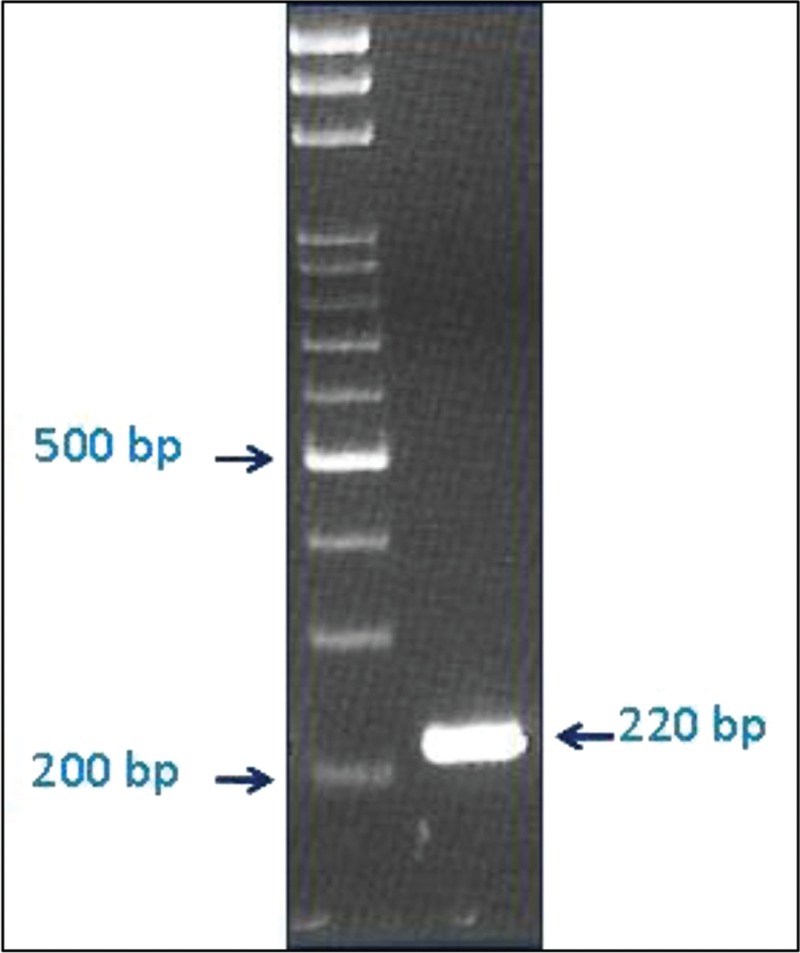
Subspecies identification with Ft-M19 assay, the PCR product has a band over 220 bp area. PCR = polymerase chain reaction.

A blood culture was performed in 2 sets, and both sets were positive for gram-negative bacilli after 1 day. A polymerase chain reaction (PCR) analysis for the 16S rRNA showed the presence of *F tularensis* on detection of the lpnA encoding a 17-kDa lipoprotein which is specific for strains of the genus *Francisella*.^4^ Using the primer of 5-AGGCGGAGATCTAGGAA-CCTTT-3 and 5-AGCCCAAGCTGACTAAAATCTTT-3, *F tularensis* subsp. *holarctica* was confirmed by the amplification of Ft-M19, which is located between the genes deaD and ppiC, showing the specific 30-bp deletion (Figure 3).^5^ The serology test for tularemia by the capture enzyme-linked immunosorbent assay, which was based on antigen binding to a monoclonal antibody (mAb) coated onto a solid phase (microtitre plate) and detection of the bound antigen using the same mAb (peroxidase labeled), had been prescribed but were negative result at that time.^6^ Sputum, bronchial washing, and pleural effusion cultures were negative for bacteria, fungi, and tuberculosis. In addition, tests for Mycoplasma, Legionella, and human immunodeficiency virus were negative. Streptomycin (1 g every 12 h) and doxycycline (100 mg every 12 h) were administered for severe tularemia with bacteremic pneumonitis. The fever subsided after 1 day, and parenchymal changes in the lung improved steadily thereafter. Streptomycin was administered for 10 days, and doxycycline, for 2 weeks. Although no pathogen was isolated from the lung or associated specimen, the clinical course of concomitant bacteremia and fulminant pneumonia suggested that they were caused by the same pathogen of *F tularensis*. The fever, pneumonia, and pleural effusion were progressive at first even under coverage with extended-spectrum antibiotics. All were resolved with appropriate antibiotics under guidance of the microbiological evidence.

Assessment of risk exposures regarding this case revealed no history of hunting, skinning, farming, lawn mowing, brush cutting, or contaminated food (such as hares) consumption. The patient is a biochemist who oversees his own biochemistry company, which manufactures products from the ascites of mice and the serum of rabbits. During the past 6 years, he was in frequent contact with the secretion of the animals (either ascites or serum) during routine laboratory procedures. Aerosol inhalation might be the route of transmission of pneumonic tularemia.

## DISCUSSIONS

Tularemia is a zoonotic infection primarily observed in the Northern Hemisphere, and it is caused by *F tularensis*, a strictly aerobic, Gram-negative coccobacillus. It is associated with rodents and hares and is transmitted to humans by direct contact with animals, aerogenic exposure, contaminated food or water consumption, and arthropod bites. Laboratory-acquired tularemia has been frequently reported,^7^ and it remains a threat for laboratory personnel. The most common (80% of cases) clinical manifestation of tularemia is ulceroglandular tularemia. Pneumonia occurs in approximately 30% of patients with ulceroglandular tularemia and 80% of those with typhoidal tularemia (prevalence varies widely between clinical series).^1^

*F tularensis* has two predominant subspecies: subsp *tularensis* (type A) and subsp. *holarctica* (type B). Type A is predominantly isolated in North America, highly virulent, and can cause invasive diseases such as pneumonia and bacteremia. Before the advent of effective antibiotics, the mortality rate of tularemia was 5% to 10%. Type B spreads more widely over the Northern Hemisphere, and it is the predominant subspecies in Europe, Japan, and North America. Type B is much less virulent compared with type A.^2^ It is rarely invasive and almost never causes lethal infection in humans, even when early appropriate treatment is not applied.^8,9^

Primary pneumonia is associated with pulmonary involvement because of inhalation, whereas secondary pneumonia is considered to be caused by hematogenic spread during the course of the disease.^3^ Bacteremic tularemia is rare, and most cases were due to type A.^10^ By contrast, pulmonary involvement with type B infection has been of much lower incidence, even in outbreaks or epidemics.^11,12^ Type B tularemia with bacteremia was reported only in immune-compromised hosts, including those with acquired immune deficiency syndrome, and in patients who have undergone liver transplantation.^9^

Serology is the most frequently ordered diagnostic test for *F tularensis* infection; however, it is limited by poor sensitivity because of undetectable antibody levels until the second week of illness.^13^ Blood cultures remain useful for an early diagnosis of severe forms of tularemia, whereas serological tests are not yet positive. Molecular differences have been exploited for developing PCR subtyping assays. Molecular subtyping assays are directed against an upstream region of an ribonucleic acid (Ribonucleic acid) helicase (the Ft-M19 assay), the presence or absence of the *ISFtu2* insertion sequence element (ISFtu2 assay), the *F tularensis* region of difference 1 (RD1 assay), and the *pdpD* gene (pdpD-2 assay).^5,14^

## CONCLUSIONS

We report an immune-compromised patient who presented with severe bacteremic tularemic pneumonia caused by low virulence *F tularensis* (type B) and was administered high doses of steroids. This case suggests that low virulence *F tularensis* should be included in the differential diagnoses of bacteremic pneumonia for endemic tularemia. Real-time PCR can facilitate the early diagnosis of tularemia, particularly in patients with less frequent clinical manifestations and those from endemic regions.
